# Predictors of COVID-19 vaccine acceptability among refugees and other migrant populations: A systematic scoping review

**DOI:** 10.1371/journal.pone.0292143

**Published:** 2024-07-05

**Authors:** Yasaman Yazdani, Poojitha Pai, Shahab Sayfi, Arash Mohammadi, Saber Perdes, Denise Spitzer, Gabriel E. Fabreau, Kevin Pottie

**Affiliations:** 1 Department of Family Medicine, Epidemiology and Biostatistics, Western University, London, Ontario, Canada; 2 Department of Medicine, Schulich School of Medicine and Dentistry, Western University, London, Ontario, Canada; 3 Department of Biology, Faculty of Science, University of Ottawa, Ottawa, Canada; 4 Michael G. DeGroote Cochrane Canada and GRADE Centres, Department of Health Research Methods, Evidence and Impact, McMaster University, Hamilton, Ontario, Canada; 5 Department of Family Medicine, Western University, London, Ontario, Canada; 6 Nezarat Consulting Ltd, Ottawa, Ontario, Canada; 7 School of Public Health, University of Alberta, Edmonton, Alberta, Canada; 8 Departments of Medicine and Community Health Sciences, University of Calgary, Calgary, Alberta, Canada; University of Macerata: Universita degli Studi di Macerata, ITALY

## Abstract

**Objective:**

This study aimed to map the existing literature to identify predictors of COVID-19 vaccine acceptability among refugees, immigrants, and other migrant populations.

**Methods:**

A systematic search of Medline, Embase, Scopus, APA PsycInfo and Cumulative Index of Nursing and Allied Health Literature (CINAHL) was conducted up to 31 January 2023 to identify the relevant English peer-reviewed observational studies. Two independent reviewers screened abstracts, selected studies, and extracted data.

**Results:**

We identified 34 cross-sectional studies, primarily conducted in high income countries (76%). Lower vaccine acceptance was associated with mistrust in the host countries’ government and healthcare system, concerns about the safety and effectiveness of COVID-19 vaccines, limited knowledge of COVID-19 infection and vaccines, lower COVID-19 risk perception, and lower integration level in the host country. Female gender, younger age, lower education level, and being single were associated with lower vaccine acceptance in most studies. Additionally, sources of information about COVID-19 and vaccines and previous history of COVID-19 infection, also influence vaccine acceptance. Vaccine acceptability towards COVID-19 booster doses and various vaccine brands were not adequately studied.

**Conclusions:**

Vaccine hesitancy and a lack of trust in COVID-19 vaccines have become significant public health concerns within migrant populations. These findings may help in providing information for current and future vaccine outreach strategies among migrant populations.

## Introduction

The COVID-19 pandemic poses a significant threat to public health, not only in terms of its impact on mortality and morbidity, but also due to the social and economic disruptions it has caused, as well as the burden of public health restrictions [1]. To combat this, one of the most crucial prevention strategies is widespread vaccination among the population [2].

In addition to barriers to equitable access to COVID-19 vaccines, vaccine hesitancy compounds the challenges faced, increasing the difficulties to achieve widespread vaccination coverage. Vaccine hesitancy is defined as the delay or refusal of vaccination despite its availability by the WHO Strategic Advisory Group of Experts on Immunization (SAGE) working group on vaccine hesitancy [3].

Understanding the factors that contribute to vaccine hesitancy is a complex and context-specific task. For COVID-19 vaccines, skepticism has arisen due to the novelty of the disease and concerns about their safety and efficacy. These concerns have been amplified by the prevalence of false or misleading information, or misinformation, leading to what the WHO has labeled an "infodemic" [4].

Previous research indicates that certain populations, including Refugee, Immigrant, and Migrant (RIM) populations, are at higher risk of vaccine hesitancy [5]. While there is limited research on the determinants of vaccine hesitancy in these groups, studies have shown that lack of accessibility, mistrust stemming from culturally insensitive healthcare practices, and discrimination experienced when seeking care are common reasons [6]. Furthermore, RIM populations face elevated risks of COVID-19 exposure due to their overrepresentation in high-risk occupations, crowded living conditions, social deprivation, and barriers to accessing reliable information on preventive measures [7]. Identifying predictors of COVID-19 vaccine acceptance in RIM populations could inform strategies to overcome potential obstacles and ensure effective vaccination campaigns.

### Research objectives

We conducted a systematic scoping review using an existing conceptual framework to map the English literature on COVID-19 vaccine acceptability predictors among RIM populations, and to identify the current gaps in the literature.

## Materials and methods

In this review, we applied the enhanced version of the Joanna Briggs Institute (JBI) scoping review guidance [8].

### Ethics statement

This Scoping review is exempt from the research ethics review as it is based on peer reviewed published works.

### Population, Concept, and Context

Our population of interest were refugees and other migrant populations. The International Organization for Migration (IOM) defines a migrant as “A person who moves away from his or her place of usual residence” [9]. Our study focused on predictors of COVID-19 vaccine acceptability among international migrants. A list of the relevant terms is available in Table 1.

**Table 1:** Population, Concept, and Context (PCC) and relevant keywords.

PCC elements:	Search terms	Medline Search results
**Population (Refugees and other migrant populations)**	Immigrant, migrant, emigrant, refugee, asylum seeker, diaspora, newcomer, foreigner, foreign-born, foreign worker	750
**Concept (vaccine acceptability)**	Immunization, vaccine, vaccination, vaccine hesitancy, vaccination hesitancy, vaccine refusal, anti-vaccine, hesitancy, hesitation, trust, acceptance, refusal, willingness, attitude, choice, denial, phobia, avoidance, decision, uptake, doubt, resistance, reluctance, exemption, controversy, dilemma, intention, skeptic, delay, distrust, mistrust, confidence, acceptability, perception, concern, fear, belief, dropout, rejection, behavior, knowledge	
**Context (COVID-19 pandemic and vaccination)**	Covid-19, severe acute respiratory syndrome coronavirus 2, Coronavirus, Sars-cov-2	

### Inclusion criteria

Observational, peer-reviewed studies published in English up until January 31, 2023, focusing on predictors of COVID-19 vaccine acceptability and similar concepts (vaccine hesitancy and vaccine intention) among RIM populations were eligible. Participants of all age groups were eligible, and studies from any country were included, regardless of their income class. We also included general population studies if more than 50% of their participants were immigrants or refugees, or a separate sub-group analysis of RIM were reported. Reviews were not eligible; however, the references of relevant reviews were screened in order to find any eligible original research articles.

### Exclusion criteria

Qualitative studies, editorials, and commentaries were excluded. Additionally, studies focusing solely on vaccine uptake and coverage were excluded unless they also reported information about predictors of vaccine acceptance or intention.

### Search methods

Keywords, and index terms suggested by refugee and immigrant health professionals and the Western University research librarian, helped us develop a comprehensive search strategy for the targeted databases. We searched the following databases, without any search restrictions: Medline, Embase, Scopus, APA PsycInfo and Cumulative Index of Nursing and Allied Health Literature (CINAHL). We also searched the World Health Organization (WHO), The United Nations High Commissioner for Refugees (UNHCR) and the International Organization for Migration (IOM) websites for relevant information. Our full Medline search strategy is presented in S1 Table.

### Screening and selection

Following the search, all identified publications were collated and uploaded on COVIDENCE (a web-based platform that manages systematic review data) to review and remove duplicates. All the stages of screening were done separately by two reviewers. Any conflicts between primary reviewers were resolved through discussion and getting the opinion of a third senior reviewer.

### Data extraction and management

Two reviewers extracted and charted the data, using a data extraction tool based on a previously reported conceptual framework (S2 Table) [10].

### Synthesis of the results

Finally, we presented the results according to the Preferred Reporting Items for Systematic reviews and Meta-Analyses extension for Scoping Reviews (PRISMA-ScR) S1 Checklist [11] in tabular form, accompanied by a narrative summary. As a scoping review, our study aimed to provide an overview of the existing literature rather than to assess the quality of included studies.

## Results

**Fig 1:**
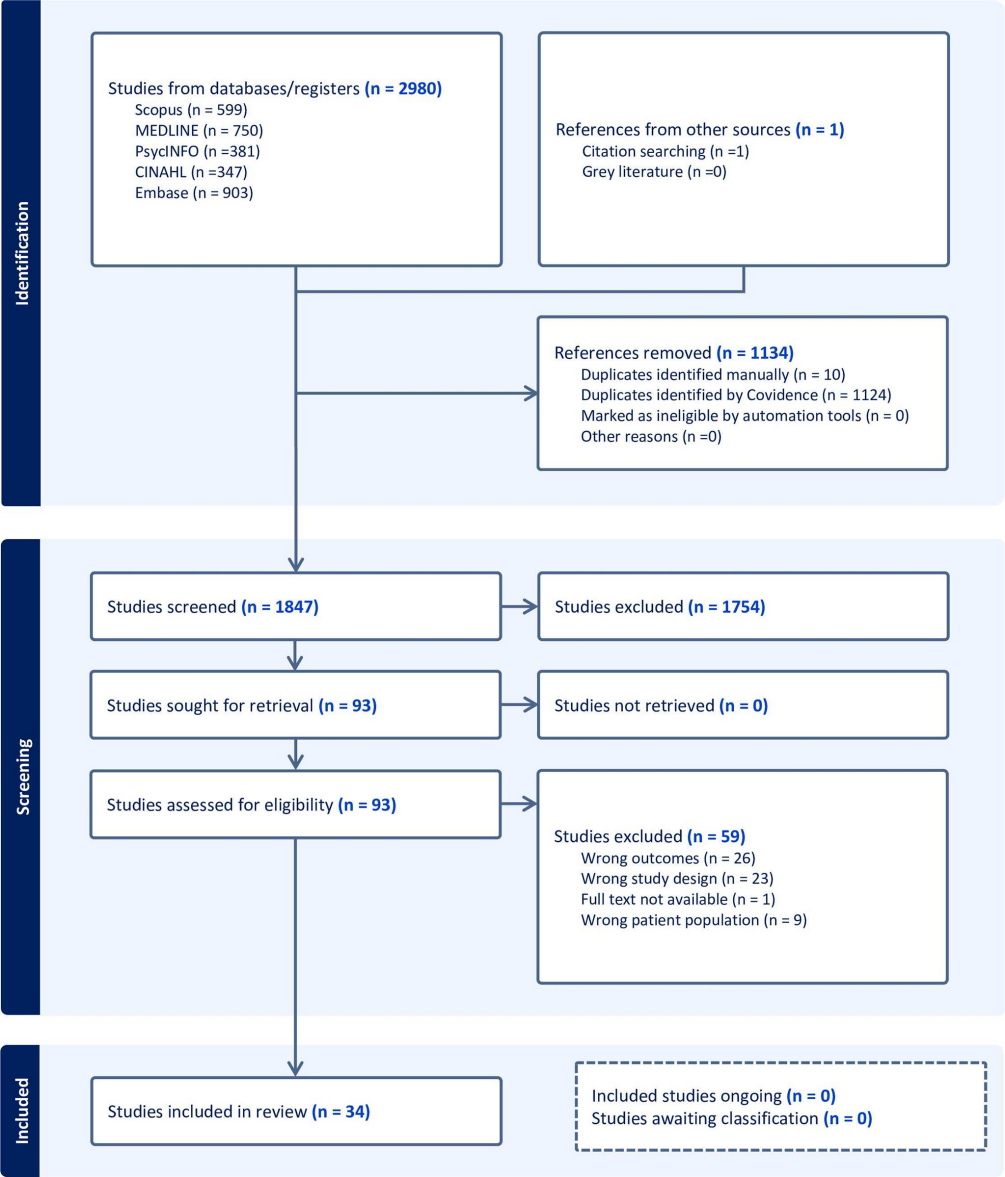
Selection of included studies (PRISMA flow diagram).

Out of the total 1847 studies screened, 34 studies were found to meet the inclusion criteria. No further relevant studies were found on WHO, IOM or UNHCR websites. Fig 1 displays the study selection process details and provides reasons for excluding articles. Common reasons for excluding articles were their focus on factors related to vaccine coverage/uptake and barriers to vaccine access, a predominant non-migrant population, and inappropriate study design.

### Characteristics of included studies

Characteristics of included studies along with the databases these studies are retrieved from are shown in Table 2. We also present a descriptive analysis of the included studies in Table 3. Most studies were conducted nationally (89%), and in high income countries (76%). Forty eight percent of all migrant participants were female and 52% were male. Eight (24%) and two (6%) articles studied refugees and undocumented migrants respectively, while one (3%) focused on international students exclusively.

**Table 2:** Characteristics of included studies.

	Study ID	Study Title	Country of Study	Target Population(s)	Objective of Study	Database
**1**	Achangwa, 2021 [12]	Acceptance of the covid-19 vaccine by foreigners in South Korea	South Korea	Foreign population	To identify the determinants of covid-19 vaccine acceptance	Medline Embase
**2**	Acharya, 2021 [13]	Assessing attitude toward COVID-19 vaccination in South Korea	South Korea	Immigrants	To assess the attitude of immigrants toward the acceptance of COVID-19 vaccines in South Korea	Medline Psych info Scopus
**3**	Aktürk, 2021 [14]	COVID-19 vaccine hesitancy in people with migratory backgrounds: a cross-sectional study among Turkish- and German-speaking citizens in Munich	Germany	People with migratory background	To assess the knowledge, attitude and behavior regarding covid-19 and vaccine intention	Medline Embase CINAHL Scopus
**4**	Alabdulla, 2021 [15]	COVID-19 vaccine hesitancy and attitudes in Qatar: A national cross-sectional survey of a migrant-majority population	Qatar	Residents of Qatar	To evaluate the degree of vaccine hesitance and its socio-demographic and attitudinal determinants	Medline Embase Scopus
**5**	Al-Hatamleh, 2022 [16]	Experiences and perceptions of COVID-19 infection and vaccination among Palestinian refugees in Jerash camp and Jordanian citizens: a comparative cross-sectional study by face-to-face interviews	Jordan	Palestinian refugees and Jordanian citizens	To assess the experiences and perceptions of COVID-19 infection and vaccination among Palestine refugees in Jerash camp compared to resident Jordanian citizens	Medline Scopus
**6**	Ali, 2022 [17]	Variations in COVID-19 Vaccine Attitudes and Acceptance among Refugees and Lebanese Nationals Pre- and Post-Vaccine Rollout in Lebanon	Lebanon	Refugees and Lebanese nationals	To assess Knowledge, attitude and perceptions regarding covid-19 vaccine factors associated with acceptance of vaccine pre- and post-vaccine roll out	Medline Embase Scopus
**7**	J.D Allen, 2022 [18]	Intention to obtain a COVID-19 vaccine among Brazilian immigrant women in the U.S	USA	Immigrant women	To assess intention to get a COVID-19 vaccine, attitudes toward vaccines, and perceptions about the pandemic	Medline Embase Scopus
**8**	K Allen, 2022 [19]	Factors associated with COVID-19 booster vaccine willingness among migrants from the Eastern Mediterranean living in Australia: a cross-sectional study	Australia	Migrants living in Australia born in the World Health Organization’s Eastern Mediterranean Region (EMRO).	To assess the factors associated with willingness to receive a COVID-19 booster vaccine among migrants living in Australia born in the World Health Organization’s Eastern Mediterranean Region (EMRO)	Medline CINAHL Scopus
**9**	Contreras-Perez, 2022 [20]	Preliminary Analysis of COVID-19 Vaccination Factors among Native and Foreign-Born Hispanic/Latine Adults Residing in South Florida, U.S.A	USA	Hispanic/Latino adults	To identify motivators, barriers and trusted sources of information influencing intention to get vaccinated	Medline Embase Scopus
**10**	Frisco, 2022 [21]	Racial/ethnic and nativity disparities in U.S. Covid-19 vaccination hesitancy during vaccine rollout and factors that explain them	USA	U.S.- and foreign-born Hispanic, black and white adults	To assess racial/ethnic and nativity disparities that affect vaccine hesitancy	Medline Embase CINAHL Scopus
**11**	Führer, 2022 [22]	COVID-19 Vaccine Acceptance and Its Determinants among Migrants in Germany-Results of a Cross-Sectional Study	Germany	People with migratory background	To assess barriers to access and general attitude towards vaccination	Medline Embase
**12**	Hnuploy, 2022 [23]	COVID-19 Vaccine Acceptance and Its Determinants among Myanmar Migrant Workers in Southern Thailand	Thailand	Myanmar Migrant Workers	To report on the willingness to obtain COVID-19 immunization and the factors related to its acceptance among Myanmar migrant workers in southern Thailand	Medline Embase Scopus
**13**	Holz, 2022 [24]	How Does Migration Background Affect COVID-19 Vaccination Intentions? A Complex Relationship Between General Attitudes, Religiosity, Acculturation and Fears of Infection	Germany	People with and without migratory background	To investigate the relationship between migration background and COVID-19 vaccine intentions	Medline Embase Scopus
**14**	Khaled, 2021 [25]	Prevalence and potential determinants of covid-19 vaccine hesitancy and resistance in Qatar: Results from a nationally representative survey of Qatari nationals and migrants between December 2020 and January 2021	Qatar	Qatari nationals and blue/white collar migrants	To estimate the prevalence and identify potential determinants of COVID-19 vaccine willingness	Medline Embase Scopus
**15**	Kheil, 2022 [26]	COVID-19 Vaccine Hesitancy among Arab Americans	USA	Arab immigrants in USA	To identify reasons for vaccine hesitancy	Medline Embase Scopus
**16**	Kitro, 2021 [27]	Acceptance, attitude, and factors affecting the intention to accept COVID-19 vaccine among Thai people and expatriates living in Thailand	Thailand	Thai nationals and expatriates living in Thailand	To assess acceptance, attitude, and determinants for COVID-19 vaccination among Thai people and expatriates living in Thailand.	Embase Scopus
**17**	Lajunen, 2022 [28]	Acculturation, trust to health care system, and attitudes to COVID-19 vaccination: A comparative study between Polish immigrants in Norway, Polish in Poland, and Norwegians in Norway	Norway	Polish immigrants in Norway, Polish in Poland, and Norwegians in Norway	To assess the relationships between acculturation to Norwegian culture, trust in health authorities, and attitude to COVID-19 vaccine	Medline Embase
**18**	Liddell, 2021 [29]	Factors associated with COVID-19 vaccine hesitancy amongst refugees in Australia	Australia	Refugees and asylum seekers	To assess perceived barrier to vaccination and predictors of vaccine hesitancy	Medline Embase Scopus
**19**	Lin, 2022 [30]	COVID-19 Pandemic and Im/migrants’ Elevated Health Concerns in Canada: Vaccine Hesitancy, Anticipated Stigma, and Risk Perception of Accessing Care	Canada	Canadian-born and immigrants in Canada	To characterize three COVID- 19-related health concerns (i.e., vaccine hesitancy, anticipated stigma, and risk perception) in Canada and how they differ based on im/migration status and other social determinants	Medline Embase CINAHL Scopus
**20**	Martinez-Donate, 2022 [31]	COVID-19 testing, infection, and vaccination among deported Mexican migrants: Results from a survey on the Mexico-U.S. border	USA	Mexican migrants deported from the U.S.	To assess COVID-19 testing, infection, treatment and vaccination rates among Mexican migrants deported from the U.S.	Medline Embase Scopus
**21**	Miner, 2023 [32]	Acceptance of COVID-19 vaccine among sub-Saharan Africans (SSA): a comparative study of residents and diasporan dwellers	Sub Saharan countries	Sub Saharan African (local residents and diaspora)	To compare the uptake of, resistance and hesitancy to the COVID-19 vaccine between SSA locally residents and in the diasporan dwellers	Medline Scopus
**22**	Ogunbajo, 2022 [33]	Acceptability of COVID-19 vaccines among Black immigrants living in the United States	USA	First and second-generation black immigrants	To examine acceptability of COVID vaccine among black immigrants in USA	Medline Embase Scopus
**23**	Page, 2022 [34]	COVID-19 vaccine hesitancy among undocumented migrants during the early phase of the vaccination campaign: A multicentric cross-sectional study	USA, Italy, France, Switzerland	Undocumented migrants	To explore undocumented migrants’ hesitancy about COVID-19 vaccine	Medline Embase Scopus
**24**	Reagu, 2023 [35]	COVID-19 Vaccine Hesitancy and Personality Traits; Results from a Large National Cross-Sectional Survey in Qatar	Qatar	Residents of Qatar	To assess the association of personality domains and vaccine hesitancy	Medline
**25**	Rego, 2022 [36]	COVID-19 vaccine hesitancy among non-refugees and refugees in Kenya	Kenya	Refugees and non-refugees	To investigate factors associated with vaccine hesitancy among refugee populations	Medline
**26**	Salibi, 2021 [37]	COVID-19 vaccine acceptance in older Syrian refugees: Preliminary findings from an ongoing study	Lebanon	Older Syrian refugees	To assess vaccine intention among a sample of older Syrian refugees in Lebanon	Medline Embase Scopus
**27**	Seo, 2022 [38]	International migrants and coronavirus disease 2019 vaccinations: social media, motivated information management, and vaccination willingness	USA	International migrants	To examine how demographic characteristics are associated with perceived uncertainty related to COVID-19 vaccinations and exposure to online misinformation about the topic	Medline
**28**	Shaw, 2022 [39]	COVID-19 vaccination intention and behavior in a large, diverse, U.S. refugee population	USA	Refugees	To assess vaccination attitude, needs and barriers	Medline Embase Scopus
**29**	Sudhinaraset, 2022 [40]	Immigration enforcement exposures and COVID-19 vaccine intentions among undocumented immigrants in California	USA	Undocumented migrants	To assess undocumented immigrants’ vaccine intentions and associations with exposure to the immigration enforcement system	Medline Embase Scopus
**30**	Talafha, 2022 [41]	COVID-19 Vaccine Acceptance among Vulnerable Groups: Syrian Refugees in Jordan	Jordan	Refugees in camp	To assess willingness of Syrian refugees in Jordan to take the COVID-19 vaccine	Medline Embase Scopus
**31**	Teng, 2022 [42]	Does the Integration of Migrants in the Host Society Raise COVID-19 Vaccine Acceptance? Evidence From a Nationwide Survey in Japan	Japan	Migrants	To explore migrants’ intention to get COVID vaccination and their reasons, and the association between integration and vaccine acceptance	Medline Embase CINAHL Scopus
**32**	Walker, 2021 [43]	Vaccine Acceptance and Its Influencing Factors: An Online Cross-Sectional Study among International College Students Studying in China	China	International college students	To identify the factors influencing covid-19 vaccine acceptance	Citation search
**33**	West, 2021 [44]	COVID-19 Vaccine Hesitancy among Temporary Foreign Workers from Bangladesh	Saudi Arabia UAE Oman, Qatar, Bahrain and Kuwait Singapore or Malaysia Europe or N. America	Temporary foreign workers	To understand variations in vaccine hesitancy among individuals in a single migrant source population across different destinations and understand the influence of the context of immigration on hesitancy	Embase
**34**	Zhang, 2021 [45]	Acceptance of COVID-19 Vaccine Among Refugees in the United States	United States	Refugees	To measure COVID-19 vaccination intentions among a sample of refugees in the United States and the reasons for their vaccine acceptance or hesitancy	Medline Embase Scopus

**Table 3:** Descriptive analysis of included studies.

Study characteristics	Study attributes	Number of studies (%) N = 34	Study ID
**Host country**	**High income** **Middle income** **Low income**	26 (76%) 8 (24%) 2 (6%)	(Achangwa et al., 2021; Acharya et al., 2021; Aktürk et al., 2021; Alabdulla et al., 2021; J. D. Allen et al., 2022; K. Allen et al., 2022; Contreras-Pérez et al., 2022; Frisco et al., 2022; Führer et al., 2022; Holz et al., 2022; Khaled et al., 2021; Kheil et al., 2022; Lajunen & Wróbel, 2022; Liddell et al., 2021; Shen Lin, 2022; Martínez-Donate et al., 2022; Ogunbajo & Ojikutu, 2022; Page et al., 2022; Reagu et al., 2023; Seo et al., 2022; Shaw et al., 2022; Sudhinaraset et al., 2022; Teng et al., 2023; Walker et al., 2021; West et al., 2021; Zhang et al., 2021) (Al-Hatamleh et al., 2022; Ali et al., 2022; Hnuploy et al., 2022; Kitro et al 2021; Miner et al., 2023 ; Salibi et al., 2021; Talafha et al., 2022; West et al., 2021) (Miner et al., 2023 ; Rego et al., 2022)
	**USA** **Germany** **South Korea** **Australia** **Qatar** **Thailand** **Lebanon** **other**	11 (32%) 3 (9%) 2 (6%) 2(6%) 3 (9%) 2 (6%) 2 (6%) 10 (29%)	(J. D. Allen et al., 2022; Contreras-Pérez et al., 2022; Frisco et al., 2022; Kheil et al., 2022; Martínez-Donate et al., 2022; Ogunbajo & Ojikutu, 2022; Page et al., 2022; Seo et al., 2022; Shaw et al., 2022; Sudhinaraset et al., 2022; Zhang et al., 2021) (Aktürk et al., 2021; Führer et al., 2022; Holz et al., 2022) (Achangwa et al., 2021; Acharya et al., 2021) (K. Allen et al., 2022; Liddell et al., 2021) (Alabdulla et al., 2021; Khaled et al., 2021; Reagu et al., 2023) (Hnuploy et al., 2022; Kitro et al., 2021) (Ali et al., 2022; Salibi et al., 2021) (Lajunen & Wróbel, 2022; Liddell et al., 2021; Shen Lin, 2022; Miner et al., 2023; Page et al., 2022; Rego et al., 2022; Talafha et al., 2022; Teng et al., 2023; Walker et al., 2021; West et al., 2021)
**Geographical coverage**	**National** **International**	30 (89%) 4 (11%)	(Achangwa et al., 2021; Acharya et al., 2021; Aktürk et al., 2021; Al-Hatamleh et al., 2022; Alabdulla et al., 2021; Ali et al., 2022; J. D. Allen et al., 2022; K. Allen et al., 2022; Contreras-Pérez et al., 2022; Frisco et al., 2022; Führer et al., 2022; Hnuploy et al., 2022; Holz et al., 2022; Khaled et al., 2021; Kheil et al., 2022; Kitro et al., 2021; Liddell et al., 2021; Shen Lin, 2022; Martínez-Donate et al., 2022; Ogunbajo & Ojikutu, 2022; Reagu et al., 2023; Rego et al., 2022; Salibi et al., 2021; Seo et al., 2022; Shaw et al., 2022; Sudhinaraset et al., 2022 ; Talafha et al., 2022; Teng et al., 2023; Walker et al., 2021; Zhang et al., 2021) (Lajunen & Wróbel, 2022 ; Miner et al., 2023; Page et al., 2022; West et al., 2021)
**Study design**	**Cross sectional** **Mixed method**	34 (100%) 2 (6%)	(Achangwa et al., 2021; Acharya et al., 2021; Aktürk et al., 2021; Al-Hatamleh et al., 2022; Alabdulla et al., 2021; Ali et al., 2022; J. D. Allen et al., 2022; K. Allen et al., 2022; Contreras-Pérez et al., 2022; Frisco et al., 2022; Führer et al., 2022; Hnuploy et al., 2022; Holz et al., 2022; Khaled et al., 2021; Kheil et al., 2022; Kitro et al., 2021; Lajunen & Wróbel, 2022; Liddell et al., 2021; Shen Lin, 2022; Martínez-Donate et al., 2022; Miner et al., 2023; Ogunbajo & Ojikutu, 2022; Page et al., 2022; Reagu et al., 2023; Rego et al., 2022; Salibi et al., 2021; Seo et al., 2022; Shaw et al., 2022; Sudhinaraset et al., 2022; Talafha et al., 2022; Teng et al., 2023; Walker et al., 2021; West et al., 2021; Zhang et al., 2021) (Seo et al., 2022; Shaw et al., 2022)
**Sampling techniques**	**Convenience sampling** **Snowball sampling** **Probability sampling** **Not mentioned**	12 (35%) 4 (12%) 6 (18%) 12 (35%)	(Acharya et al., 2021; Alabdulla et al., 2021; Ali et al., 2022; J. D. Allen et al., 2022; K. Allen et al., 2022; Führer et al., 2022; Kitro et al., 2021; Lajunen & Wróbel, 2022; Liddell et al., 2021; Reagu et al., 2023; Shaw et al., 2022; Talafha et al., 2022) (Achangwa et al., 2021; Aktürk et al., 2021; Ogunbajo & Ojikutu, 2022; Zhang et al., 2021) (Frisco et al., 2022; Hnuploy et al., 2022; Khaled et al., 2021; Shen Lin, 2022; Martínez-Donate et al., 2022; Walker et al., 2021) (Al-Hatamleh et al., 2022; Contreras-Pérez et al., 2022; Holz et al., 2022; Kheil et al., 2022; Miner et al., 2023; Page et al., 2022; Rego et al., 2022; Salibi et al., 2021; Seo et al., 2022; Sudhinaraset et al., 2022; Teng et al., 2023; West et al., 2021)
**Sample size**	**Range** **Less than 500** **500–1000** **More than 1000**	(127–7821) 17 (50%) 6 (18%) 11 (32%)	(Acharya et al., 2021; Aktürk et al., 2021; Al-Hatamleh et al., 2022; J. D. Allen et al., 2022; K. Allen et al., 2022; Contreras-Pérez et al., 2022; Führer et al., 2022; Khaled et al., 2021; Martínez-Donate et al., 2022; Ogunbajo & Ojikutu, 2022; Seo et al., 2022; Shaw et al., 2022; Sudhinaraset et al., 2022; Talafha et al., 2022; Walker et al., 2021; West et al., 2021; Zhang et al., 2021) (Achangwa et al., 2021; Khaled et al., 2021; Kitro et al., 2021; Lajunen & Wróbel, 2022; Liddell et al., 2021; Page et al., 2022) (Alabdulla et al., 2021; Ali et al., 2022; Frisco et al., 2022; Holz et al., 2022; Kheil et al., 2022; Shen Lin, 2022; Miner et al., 2023; Reagu et al., 2023; Rego et al., 2022; Salibi et al., 2021; Teng et al., 2023)
**Gender**	**Male** **Female**	20412(52%) 18630(48%)	
**Other demographic characteristics**	**Racialized status/ethnicity** **Immigration status** **Region of origin**	11 (32%) 16 (47%) 19 (56%)	(Achangwa et al., 2021; Acharya et al., 2021; Alabdulla et al., 2021; J. D. Allen et al., 2022; Contreras-Pérez et al., 2022; Frisco et al., 2022; Khaled et al., 2021; Kheil et al., 2022; Ogunbajo & Ojikutu, 2022; Shaw et al., 2022; Sudhinaraset et al., 2022) (Al-Hatamleh et al., 2022; Contreras-Pérez et al., 2022; Führer et al., 2022; Hnuploy et al., 2022; Khaled et al., 2021; Kheil et al., 2022; Martínez-Donate et al., 2022; Page et al., 2022; Rego et al., 2022; Salibi et al., 2021; Shaw et al., 2022; Sudhinaraset et al., 2022; Talafha et al., 2022; Walker et al., 2021; West et al., 2021; Zhang et al., 2021) (Aktürk et al., 2021; Ali et al., 2022; J. D. Allen et al., 2022; K. Allen et al., 2022; Führer et al., 2022; Hnuploy et al., 2022; Holz et al., 2022; Kheil et al., 2022; Kitro et al., 2021; Martínez-Donate et al., 2022; Miner et al., 2023; Page et al., 2022; Salibi et al., 2021; Seo et al., 2022; Shaw et al., 2022; Talafha et al., 2022; Walker et al., 2021; West et al., 2021; Zhang et al., 2021)
**Target population**	**Refugees** **Undocumented migrants** **Migrant workers** **International students** **Any type of migratory background/ status** **Migrants as a subgroup of general population**	8 (24%) 2 (6%) 2 (6%) 1 (3%) 9 (26%) 16 (47%)	(Al-Hatamleh et al., 2022; Ali et al., 2022; Liddell et al., 2021; Rego et al., 2022; Salibi et al., 2021; Shaw et al., 2022; Talafha et al., 2022; Zhang et al., 2021) (Page et al., 2022; Sudhinaraset et al., 2022) (Hnuploy et al., 2022; West et al., 2021) (Walker et al., 2021) (Achangwa et al., 2021; Acharya et al., 2021; J. D. Allen et al., 2022; K. Allen et al., 2022; Führer et al., 2022; Martínez-Donate et al., 2022; Miner et al., 2023; Reagu et al., 2023; Seo et al., 2022; Teng et al., 2023) (Aktürk et al., 2021; Al-Hatamleh et al., 2022; Alabdulla et al., 2021; Ali et al., 2022; Contreras-Pérez et al., 2022; Frisco et al., 2022; Holz et al., 2022; Khaled et al., 2021; Kheil et al., 2022; Kitro et al., 2021; Lajunen & Wróbel, 2022; Shen Lin, 2022; Miner et al., 2023; Ogunbajo & Ojikutu, 2022; Reagu et al., 2023; Rego et al., 2022)
**Timeline**	**Before WHO first COVID-19 vaccination recommendation** **After WHO first COVID-19 vaccination recommendation**	4 (12%) 30 (88%)	(Alabdulla et al., 2021; J. D. Allen et al., 2022; Shen Lin, 2022; Reagu et al., 2023) (Achangwa et al., 2021; Acharya et al., 2021; Aktürk et al., 2021; Al-Hatamleh et al., 2022; Ali et al., 2022; K. Allen et al., 2022; Contreras-Pérez et al., 2022; Frisco et al., 2022; Führer et al., 2022; Hnuploy et al., 2022; Holz et al., 2022; Khaled et al., 2021; Kheil et al., 2022; Kitro et al., 2021; Lajunen & Wróbel, 2022; Liddell et al., 2021; Martínez-Donate et al., 2022; Miner et al., 2023; Ogunbajo & Ojikutu, 2022; Page et al., 2022; Rego et al., 2022; Salibi et al., 2021; Seo et al., 2022; Shaw et al., 2022; Sudhinaraset et al., 2022; Talafha et al., 2022; Teng et al., 2023; Walker et al., 2021; West et al., 2021; Zhang et al., 2021)
**Vaccine outcomes**	**Vaccine acceptance** **Vaccine hesitancy** **Vaccine intention**	9 (26%) 11 (32%) 14 (42%)	(Achangwa et al., 2021; Acharya et al., 2021; Hnuploy et al., 2022; Kitro et al., 2021; Lajunen & Wróbel, 2022; Ogunbajo & Ojikutu, 2022; Talafha et al., 2022; Walker et al., 2021; Zhang et al., 2021) (Al-Hatamleh et al., 2022; Frisco et al., 2022; Khaled et al., 2021; Kheil et al., 2022; Liddell et al., 2021; Shen Lin, 2022; Miner et al., 2023; Page et al., 2022; Reagu et al., 2023; Rego et al., 2022; West et al., 2021) (Aktürk et al., 2021; Alabdulla et al., 2021; Ali et al., 2022; J. D. Allen et al., 2022; K. Allen et al., 2022; Contreras-Pérez et al., 2022; Holz et al., 2022; Martínez-Donate et al., 2022; Salibi et al., 2021; Seo et al., 2022; Shaw et al., 2022; Sudhinaraset et al., 2022; Teng et al., 2023; Zhang et al., 2021)
	**Hesitancy towards booster dose**	2 (6%)	(K. Allen et al., 2022; Kheil et al., 2022)
	**Acceptability based on vaccine brand/country of origin**	3 (9%)	(Ali et al., 2022; Kitro et al., 2021; Talafha et al., 2022)

Ninety percent of studies were conducted after the first WHO recommendation regarding COVID-19 vaccination, released in December 2020. Only two studies (6%) examined migrants’ hesitancy towards getting COVID-19 booster dose and three (9%) evaluated participants’ preferences regarding different vaccine brands offered or their country of origin. No information was provided regarding whether the type of vaccine provided for immigrants and refugees differs from that given to the general population and whether this distinction contributes to hesitancy. In the study by Kitro et al., the majority of respondents expressed a preference for imported vaccines, particularly those from the US [27]. However, in Talafha et al.’s study, most participants indicated that their comfort with vaccines was not contingent on whether they were developed in America/Europe versus other regions [41]. In Ali et al.’s study, some participants mentioned the possibility of accepting a specific vaccine while rejecting another; however, the study did not provide information about their preferred types of vaccines [17].

The variables examined in different studies are reported in S3 Table. However, only some studies investigated the association of these factors with vaccine acceptance/intention/hesitancy (Table 4). According to the literature review and based on the conceptual framework used [10], we categorized predictors of COVID-19 vaccine acceptance into four major groups:

- Sociodemographic factors
- Communication-related factors
- COVID-19 vaccine- related factors
- Covid-19 infection related factors

**Table 4:** COVID-19 vaccine acceptance associated factors (n = number of studies examined the association between vaccine acceptance and each factor).

Study factors		Number of studies reported significant association (%)	Study ID
**Sociodemographic factors**	**Age (n = 28)** **Gender (n = 27)** **Education (n = 23)** **Marital status (n = 13)**	15 (54%) 12 (44%) 13 (57%) 4 (31%)	(Aktürk et al., 2021; Al-Hatamleh et al., 2022; Alabdulla et al., 2021; Ali et al., 2022; J. D. Allen et al., 2022; K. Allen et al., 2022; Contreras-Pérez et al., 2022; Frisco et al., 2022; Kheil et al., 2022; Kitro et al., 2021; Liddell et al., 2021; Shen Lin, 2022; Martínez-Donate et al., 2022; Miner et al., 2023; Page et al., 2022; Rego et al., 2022) (Achangwa et al., 2021; Aktürk et al., 2021; Al-Hatamleh et al., 2022; Alabdulla et al., 2021; Ali et al., 2022; Frisco et al., 2022; Holz et al., 2022; Khaled et al., 2021; Ogunbajo & Ojikutu, 2022; Reagu et al., 2023; Sudhinaraset et al., 2022; Zhang et al., 2021) (Achangwa et al., 2021; Aktürk et al., 2021; Al-Hatamleh et al., 2022; K. Allen et al., 2022; Frisco et al., 2022; Holz et al., 2022; Shen Lin, 2022; Ogunbajo & Ojikutu, 2022; Reagu et al., 2023; Rego et al., 2022; Seo et al., 2022; Sudhinaraset et al., 2022; Teng et al., 2023) (Achangwa et al., 2021; Acharya et al., 2021; Alabdulla et al., 2021; Shen Lin, 2022)
**Communication- related factors**	**Trust in authorities (n = 8)**	5 (63%)	(K. Allen et al., 2022; Frisco et al., 2022; Lajunen & Wróbel, 2022; Liddell et al., 2021; Rego et al., 2022)
	**Sources of news/information (n = 11)**	6 (55%)	(Achangwa et al., 2021; J. D. Allen et al., 2022; Holz et al., 2022; Liddell et al., 2021; Page et al., 2022; Shaw et al., 2022)
**COVID-19 Vaccine-related factors**	**Perceived vaccine safety/side effects (n = 13)**	10 (77%)	(Alabdulla et al., 2021; K. Allen et al., 2022; Führer et al., 2022; Khaled et al., 2021; Kheil et al., 2022; Kitro et al., 2021; Salibi et al., 2021 ; Talafha et al., 2022; West et al., 2021; Zhang et al., 2021)
	**Perceived vaccine efficacy (n = 9)**	8 (89%)	(Alabdulla et al., 2021; K. Allen et al., 2022; Führer et al., 2022; Kheil et al., 2022; Salibi et al., 2021; Talafha et al., 2022; Walker et al., 2021; Zhang et al., 2021)
	**Concerned about vaccine development (n = 3)**	3 (100%)	(Alabdulla et al., 2021; Frisco et al., 2022; Talafha et al., 2022)
**Covid-19 infection-related factors**	**COVID-19 risk perception (n = 10)**	9 (90%)	(Ali et al., 2022; K. Allen et al., 2022; Führer et al., 2022; Hnuploy et al., 2022; Holz et al., 2022; Khaled et al., 2021; Liddell et al., 2021; West et al., 2021; Zhang et al., 2021)
	**History/ exposure to covid-19 in participants or their relations (n = 11)**	4 (36%)	(Frisco et al., 2022; Ogunbajo & Ojikutu, 2022; Talafha et al., 2022 ; West et al., 2021)

### Sociodemographic factors

Most common sociodemographic factors associated with COVID-19 acceptance were gender, age, level of education, and marital status (Table 4). Female gender [12, 14–17, 21, 24, 25, 33, 35, 45], younger age [16, 17, 19, 24, 26, 27, 29–32, 34], lower level of education [14, 19, 21, 24, 30, 33, 35, 36, 40, 42], and being single [12, 15, 30] were associated with lower vaccine acceptance in most studies. Only a limited number of studies have investigated the acceptance of Covid-19 vaccines during pregnancy, with none specifically addressing breastfeeding. In the study by Akturk et al., pregnancy was categorized as “other reasons” for vaccine hesitancy [14]. Meanwhile, Ali et al. found that a majority of respondents were either uncertain or opposed to the idea of administering the vaccine to pregnant women [17].

Some of the other sociodemographic influencers of COVID-19 vaccine acceptance were sexual orientation [33], income [23, 30], region of origin [12, 24, 45], religion [12, 24], migratory background [13, 35, 36, 44], employment status [15, 30, 35], years living in host countries [19, 28, 31, 42, 44], racialized status [21, 40], working in health care settings [27, 32, 33], language fluency [31, 44], having comorbidities [34], and having insurance [40]. In two studies, refugees living outside the refugee camps were less likely to accept COVID-19 vaccination [36, 37]. Additionally, one study examined the relationship between different personality traits and COVID-19 vaccine hesitancy. In this study higher Conscientiousness, lower Openness and Neuroticism were associated with lower vaccine acceptance [35].

### Communication-related factors

Among the studies that examined the association between trust in authorities and COVID-19 vaccine acceptance, five studies (63%) found a positive association between level of trust in government and host country’s health care system and COVID-19 vaccine acceptance [19, 21, 28, 29, 35]. Moreover, participants’ preference to get vaccinated against COVID-19 was significantly associated with their source of COVID-19 information in 55% of studies examining this association (Table 4). Additionally, participants who had more exposure to confidence-inducing news and had no exposure to concerning news regarding COVID-19 vaccines were more likely to get the booster dose [19].

### COVID-19 vaccine-related factors

Concerns regarding the safety/side effects of COVID-19 vaccine was a prominent factor associated with lower vaccine acceptability in 10 studies (77%) evaluating this association [15, 19, 22, 25–27, 37, 41, 44, 45]. Perceived vaccine efficacy was also another predictor of vaccine acceptability. Eight articles (89%) reported lower acceptance among participants who had mistrust in COVID-19 vaccines’ effectiveness [15, 19, 22, 26, 37, 41, 43, 45]. Another reason for lower vaccine acceptance was the belief that COVID-19 vaccines were developed too quickly and that the rushed pace of testing them would become problematic in the future. Being concerned about vaccine development was positively associated with vaccine hesitancy in all three studies evaluating this association [15, 21, 41]. Additionally, six studies examined the participants’ knowledge/attitude scores towards COVID-19 vaccines [13, 17, 19, 27, 28, 41]. Two studies showed that people with greater knowledge of COVID-19 vaccines had higher intention to get vaccinated [17, 19]. Non-COVID-19 vaccine refusal and negative attitude towards vaccines in general were associated with lower COVID-19 vaccine acceptance [12, 22, 34]. Also, getting the seasonal flu vaccination [15, 41] were a positive predictor of vaccine acceptance.

### COVID-19 infection-related factors

Among studies that examined the association of COVID-19 risk perception and vaccine acceptability, nine (90%) found a positive association [17, 19, 22–25, 29, 44, 45]; whereby, participants who had higher COVID-19 risk perception had higher vaccine acceptability. Only four out of the 11 studies [14, 21, 23, 25, 26, 30, 33, 36, 41, 42, 44] that evaluated the relationship between COVID-19 vaccine acceptance and previous history of exposure/infection with COVID-19 disease reported a significant association [21, 33, 41, 44]. In the study by Frisco et al. previous infection with COVID-19 was associated with higher vaccine acceptance [21], while Ogunbajo et al. suggested that previous positive COVID-19 test was higher among vaccine hesitant participants [33]. Moreover, West et al. found that vaccine acceptance was higher among workers who had more COVID-19 exposure through crowded housing [44]. Talafha et al. reported that previous COVID-19 exposure did not affect the respondents’ willingness to take the vaccine. Some studies examined the association of vaccine acceptability with knowledge and attitude regarding COVID-19 infection. All reported that higher COVID-19 infection knowledge and attitude scores were associated with higher vaccine acceptability [14, 27, 32, 41]. Participants’ health behaviors during COVID-19 pandemic were other factors measured in some studies. They found wearing a face mask and social distancing were associated with higher COVID-19 vaccine acceptance/intention [23, 27, 36].

### Other factors associated with COVID-19 vaccine hesitancy

Although concerns regarding vaccine safety and effectiveness and lower COVID-19 risk perceptions were among most common predictors of vaccine hesitancy, fear of needles [22, 45], belief in house remedies [22], religious reasons [22, 23, 26], belief in natural exposure to germs and viruses as the best protection and avoiding any interference with nature [15, 22] were other reasons for not getting the vaccine, reported by vaccine hesitant or anti vaccine individuals.

Shaw et al. reported that individuals with high vaccine acceptability had a lower social vulnerability index [39]. Another study by page et al. showed that higher immigration enforcement exposure, such as experiencing or knowing someone who experienced immigration raids, detention or deportation, was associated with lower odds of COVID-19 vaccine acceptance [34]. In contrast, acculturation and social integration of migrants were associated with higher vaccine acceptance [28, 42], as well as cues to action [17, 43] and social norms [17]. Cues to action, refers to being motivated to get vaccinated by seeing neighbors, community leaders, doctors, or politicians receive the vaccine; whereas vaccination as a social norm refers to the higher likelihood of getting vaccinated when most people an individual knew had received the vaccine.

## Discussion

Our findings suggest that predictors of COVID-19 vaccine acceptance among migrant populations can be categorized in four major groups: “sociodemographic factors”, “communication-related factors”, “COVID-19 vaccine-related factors” and “COVID-19 infection- related factors”. Lower vaccine acceptance was associated with mistrust in the host countries’ government and healthcare system, concerns about the safety and effectiveness of COVID-19 vaccines, limited knowledge of COVID-19 infection and vaccines, lower COVID-19 risk perception, and lower integration level in the host country. Also, female gender, younger age, lower education level, and being single were sociodemographic factors associated with lower vaccine acceptance in most studies. Sources of information regarding COVID-19 and vaccines and previous history of COVID-19 infection were other influencers of vaccine acceptance.

A systematic review on qualitative studies on COVID-19 vaccine hesitancy among ethnic minorities similarly identified five major themes for drivers of vaccine hesitancy among these populations: 1) Institutional mistrust, 2) Lack of confidence in vaccine and development process, 3) Lack of reliable information or messengers, 4) Complacency/perceived lack of need and 5) Structural barriers to vaccine [46].

While our findings show that most studies reported lower vaccine acceptance among immigrant women, as the association between sex/gender and COVID-19 vaccine acceptance among RIM was significant in only 44% of studies examining this association, further analytic investigation is needed on this matter. In a meta-analysis conducted by Alimoradi et al. the pooled prevalence of Covid-19 vaccine acceptance did not significantly differ according to gender in migrants and ethnic minorities [47]. Another systematic review on determinants of routine and COVID-19 vaccine uptake among RIM also reported no strong association with gender [48]. Higher skepticism among females might be even more important to achieve higher vaccination coverage, as this can directly affect children COVID-19 vaccination rate, because mothers appear less willing to vaccinate their children against COVID-19 than fathers [49, 50].

Furthermore, there is insufficient research on attitudes toward Covid-19 vaccination during pregnancy and breastfeeding among RIM populations. A systematic review and meta-analysis on the prevalence of COVID-19 vaccine acceptance among pregnant women revealed that only 49% of participants were accepting of the COVID-19 vaccine [51]. Pregnant and breastfeeding immigrant and refugee women encounter unique concerns, particularly when resettled in countries with vaccination guidelines differing from their country of origin [52]. The presence of cultural, linguistic, and informational gaps amplifies hesitancy within these vulnerable subgroups. Given their lived experiences with systemic racism and mistrust in the healthcare system, it becomes essential to tailor public health messaging specifically for them [53].

Understanding the sociodemographic predictors of vaccine acceptability can help to improve community engagement strategies to increase vaccination uptake in populations with lower vaccine acceptance, such as outreach initiatives for under-immunized ethnic groups [54]. One example is a successful program addressed low vaccination rates among women of Rohingya refugees in Cox’s Bazar, Bangladesh. Strategies included educational programs, women-only radio clubs, religious group-study sessions, and employing female vaccinators. These measures improved access to accurate information and created a comfortable environment for refugee women to get vaccinated [55].

Other sociodemographic factors of potentially great importance among migrant populations include migration status, years living in the host country, region of origin, language fluency, religion, and immigration enforcement exposure [12, 13, 18, 24, 31, 42, 45]. Evidence suggests that language barriers and cultural variations are prominent factors that can affect these populations’ intention to be immunized [12].

The term “vaccine hesitancy” might imprecisely blame individuals, while there are several fundamental factors that may lead to making this decision [56]. Our findings corroborate previous research that suggests trust in the host country’s government and public health authorities are key factors that affect migrants’ attitude towards COVID-19 vaccines and vaccination decision making [49, 56, 57]. Lack of trust among migrant communities can stem from previous experience of xenophobia, racial discrimination, and anti-migrant politics [48, 57, 58]. A qualitative systematic review of COVID-19 vaccine hesitancy by Shearn et al. showed that “feeling unheard, ignored or excluded from the healthcare system” caused institutional mistrust resulting in COVID-19 vaccine hesitancy among RIM populations [46]. Moreover, trust also plays a crucial role in acceptance of non-COVID vaccines among RIM population [57].

Previous research also suggests that source of information is an important influencer of vaccination decision making [59]; however, this factor might be more complicated among RIM populations based on different cultural and linguistic backgrounds and adaptation level within host countries. Moreover, the lack of adequate information in migrants’ primary languages often lead to reliance on community networks, traditional and social media, which might lead to confusion and inability to discern factual information from misinformation [29, 34, 38]. Similarly, in non-COVID vaccination context, the overwhelming amount of contradictory information and false claims spread through social media regarding vaccines undermines the efforts to encourage vaccine acceptance among migrants [57].

A systematic review by Romate et al. suggests that the perceived safety and effectiveness of COVID-19 vaccines as well as risk perception of COVID-19 infection is associated with both “knowledge” and “trust” factors [60]. Additionally, our study revealed that increased knowledge level about both COVID-19 infection and vaccines among migrants were associated with higher vaccine acceptability. These findings reinforce previous research that investigated these associations among general populations [61, 62]. Applying various community engagement strategies may help build trust and address knowledge gaps and misinformation among these diverse communities [63]. Previous research suggests “using high touch rather than high-tech approaches”; Involving community health workers can also ensure trustworthy sources of information and tailoring new health recommendations [56].

Our study also suggests that higher levels of migrants’ acculturation and integration in the host country is associated with higher COVID-19 vaccine acceptance [28, 42, 44]. Previous research similarly showed that migrants living in societies with lower integration policies for migrant populations experience poorer health conditions [42, 64, 65]. Strategies that facilitate migrants’ psychological, social, economic, political, navigational, and linguistic integration in a host country can, in turn affect other vaccine hesitancy related factors such as trust in authorities and misinformation [42].

The ethical dimensions surrounding COVID-19 vaccination are multifaceted, with specific considerations for vulnerable groups such as the elderly residing in nursing homes. Obtaining informed consent poses challenges given their advanced age and potential cognitive limitations. Legal frameworks in Western nations emphasize the importance of involving individuals in healthcare decisions, even when faced with psychological weaknesses. These ethical dilemmas underscore the need for systematic approaches to protect the health of individuals within these fragile communities [66].

Another marginalized group that needs more attention are people with disabilities. Research shows some of the individuals with disabilities may exhibit higher levels of vaccine hesitancy, primarily due to heightened concerns about vaccine safety compared to the perceived risks associated with COVID-19 infection [67]. Incorporating universal accessibility into vaccination initiatives, sites, and communication is crucial to address the unique needs of individuals with disabilities [68]. Tailored communication strategies, written in plain language and disseminated in accessible formats, can contribute to reducing hesitancy among individuals with disabilities [67].

### Implication for public health policy

Our findings may help inform the programs and community outreach strategies to improve uptake of COVID-19 vaccines in migrant and refugee subgroups with lower vaccine acceptance. Reducing mistrust in authorities and addressing knowledge gaps among migrant populations is crucial for improving COVID-19 vaccine acceptance and similar public health challenges.

### Limitations

It is noteworthy that we noticed some limitations in the included studies. Due to the cross-sectional design of included studies, we cannot make causal claims. While these findings can provide valuable insights into factors associated with lower COVID-19 vaccine acceptance among migrant populations, it’s important to note that the majority of studies employed non-random sampling strategies. This implies that participants may not be truly representative of migrant populations; therefore, caution should be exercised when generalizing the study findings. Further, using online platforms to recruit study participants and survey in English or the host country’s language, can skew the data towards more acculturated, connected, and less vulnerable study participants. Moreover, most studies did not consider potential confounding variables such as having comorbidities, health literacy, duration of residence in host country in their analyses. Finally, recall bias, selection bias, and social desirability bias were other issues which might have affected the included studies’ findings.

## Conclusion

The acceptability of COVID-19 vaccines among RIM is influenced by various factors, including sociodemographic characteristics, communication-related factors, COVID-19 vaccine-related factors, and COVID-19 infection-related factors. Targeted vaccination plans, community engagement strategies, and efforts to address knowledge gaps and build trust are crucial for promoting vaccine acceptance among RIM populations.

## Future studies

Evaluation of migrant population’s attitude towards the COVID-19 booster dose(s) and different types and brands of COVID-19 vaccines would be beneficial. Furthermore, there is a need for more studies on COVID-19 vaccine acceptance among specific subgroups of refugees and migrants, including pregnant and breastfeeding women, children and individual with disabilities. Conducting a systematic review with an equity lens to explore sociodemographic factors associated with vaccine acceptance could enhance our understanding and aid in identifying subpopulations of migrants with lower vaccine acceptance.

## Supporting information

S1 Checklist Preferred Reporting Items for Systematic reviews and Meta-Analyses extension for Scoping Reviews (PRISMA-ScR) checklist. (DOCX)

S1 Table Medline search strategy. (PDF)

S2 Table Data extraction tool. (PDF)

S3 Table Factors examined in included studies. (PDF)

S1 File Study’s minimal data set. (XLSX)
