# Supplementary material for: Predictors of COVID-19 vaccine acceptability among refugees and other migrant populations: A systematic scoping review
Source: PLoS One. 2024 Jul 5;19(7):e0292143. doi: 10.1371/journal.pone.0292143 (PMC11226018; doi:10.1371/journal.pone.0292143)
Supplement: S1 Table — (PDF) [file pone.0292143.s002.pdf]

S1 Table. Medline search strategy

**Medline search**	(immigrant*.tw,kf. or "Emigrants and Immigrants"/) OR ("Transients and Migrants"/ or migrant*.tw,kf.) OR emigrant*.tw,kf. OR (Refugees/ or refugee*. tw,kf.) OR “asylum seeker*”. tw,kf. OR (diaspora. tw,kf. or Human Migration/) OR newcomer*. tw,kf. OR foreigner*. tw,kf. OR foreign-born*. tw,kf. OR foreign worker*. tw,kf) OR ("undocumented immigrant*" OR undocumented immigrants/)<br><br>AND<br><br>(covid-19.tw,kf. or COVID-19/) OR covid.tw,kf. OR (Severe Acute Respiratory Syndrome.tw,kf. or severe acute respiratory syndrome/) OR (Coronavirus.tw,kf. or Coronavirus/ or Coronavirus infection/) OR (Sars-cov-2.tw,kf. or SARS-CoV-2/) OR<br><br>AND<br><br>(Immunization/ or immunization.tw,kf.) OR (vaccine.tw,kf. or Vaccines/) OR (vaccination.tw,kf. or Vaccination/) OR (Vaccine hesitancy.tw,kf. or Vaccination Hesitancy/) OR Vaccination hesitancy.tw,kf. OR (Vaccine refusal.tw,kf. or Vaccination Refusal/) OR Vaccination refusal.tw,kf. OR (anti-vaccine*.tw,kf. OR Anti-Vaccination Movement/) OR anti-vax*.tw,kf. OR hesitancy.tw,kf. OR hesitation.tw,kf. OR (trust.tw,kf. or Trust/) OR acceptance.tw,kf. OR refusal.tw,kf. OR willingness.tw,kf. OR (Attitude/ or attitude.tw,kf.) OR choice.tw,kf. OR denial.tw,kf. OR (phobia.tw,kf. or Phobic Disorders/) OR avoidance.tw,kf. OR decision.tw,kf. OR uptake.tw,kf. OR doubt.tw,kf. OR resistance.tw,kf. OR reluctance.tw,kf. OR exemption.tw,kf. OR controversy.tw,kf. OR dilemma.tw,kf. OR (intention.tw,kf. or Intention/) OR skeptic.tw,kf. OR delay.tw,kf. OR distrust.tw,kf. OR mistrust.tw,kf. OR confidence.tw,kf. OR acceptability.tw,kf. OR (perception.tw,kf. or Perception/) OR risk perception.tw,kf. OR concern.tw,kf. OR (fear.tw,kf. or Fear/) OR belief.tw,kf. OR dropout.tw,kf OR rejection.tw,kf. OR (Behavior/ or behavior.tw,kf) OR (Knowledge/ or knowledge.tw,kf.)
