# Supplementary material for: Predictors of COVID-19 vaccine acceptability among refugees and other migrant populations: A systematic scoping review
Source: PLoS One. 2024 Jul 5;19(7):e0292143. doi: 10.1371/journal.pone.0292143 (PMC11226018; doi:10.1371/journal.pone.0292143)
Supplement: S2 Table — (PDF) [file pone.0292143.s003.pdf]

S2 Table. Data extraction tool

Data	Data description
**Study reference**	Author name
**Date of publication and timeline of the study**	Publication year and recruitment time period
**Country of study**	Country where the study is conducted
**Study design and sampling method**	Type of study such as cross-sectional, cohort, etc.<br><br>Method of recruitment of participants
**Study objective**	The objective/question of the study
**Participants' total number**	Number of study participants
**Outcome assessed**	Vaccine hesitancy, acceptance, and vaccine intention
**Participants' place of origin**	Country where the migrant populations came from
**Participants' socio-demographic characteristics**	Participants' Characteristics, like gender, age and immigration status

**Communication-related factors**	Factors associated with sources of information and communication about COVID-19:<br>● Trust in authorities<br>● Sources of information/news
**COVID-19 Vaccine-related factors**	Factors associated with COVID-19 vaccine:<br>● Knowledge/attitude/beliefs towards COVID-19 vaccine<br>● Being concern about COVID-19 vaccine safety/ side effects<br>● Being concern about COVID-19 vaccine efficacy<br>● Being concern about COVID-19 vaccine development
**COVID-19 infection-related factors**	Factors associated with COVID-19 infection:<br>● Knowledge/attitude/practice regarding COVID-19 infection<br>● COVID-19 risk perception<br>● History or exposure to covid-19 disease in participants or their relations
**Limitations**	Limitations of each study
