# Supplementary material for: Predictors of COVID-19 vaccine acceptability among refugees and other migrant populations: A systematic scoping review
Source: PLoS One. 2024 Jul 5;19(7):e0292143. doi: 10.1371/journal.pone.0292143 (PMC11226018; doi:10.1371/journal.pone.0292143)
Supplement: S3 Table — (PDF) [file pone.0292143.s004.pdf]

## S3 Table. Factors examined in included studies

| Study factors | Number (%) N=34 | Study ID |
|---|---|---|
| **Demographics** | 34 (100%) | (Achangwa et al., 2021; Acharya et al., 2021; Aktürk et al., 2021; Al-Hatamleh et al., 2022; Alabdulla et al., 2021; Ali et al., 2022; J. D. Allen et al., 2022; K. Allen et al., 2022; Contreras-Pérez et al., 2022; Frisco et al., 2022; Führer et al., 2022; Hnuploy et al., 2022; Holz et al., 2022; Khaled et al., 2021; Kheil et al., 2022; Kitro et al., 2021; Lajunen & Wróbel, 2022; Liddell et al., 2021; Shen Lin, 2022; Martínez-Donate et al., 2022; Miner et al., 2023; Ogunbajo & Ojikutu, 2022; Page et al., 2022; Reagu et al., 2023; Rego et al., 2022; Salibi et al., 2021; Seo et al., 2022; Shaw et al., 2022; Sudhinaraset et al., 2022; Talafha et al., 2022; Teng et al., 2023; Walker et al., 2021; West et al., 2021; Zhang et al., 2021) |
| **Trust in authorities** | 17 (50%) | (Achangwa et al., 2021; Aktürk et al., 2021; Alabdulla et al., 2021; Ali et al., 2022; K. Allen et al., 2022; Frisco et al., 2022; Hnuploy et al., 2022; Holz et al., 2022; Kheil et al., 2022; Kitro et al., 2021; Lajunen & Wróbel, 2022; Liddell et al., 2021; Martínez-Donate et al., 2022; Ogunbajo & Ojikutu, 2022; Rego et al., 2022; Salibi et al., 2021; West et al., 2021) |
| **Sources of news/information** | 19 (56%) | (Achangwa et al., 2021; Aktürk et al., 2021; Al-Hatamleh et al., 2022; Alabdulla et al., 2021; Ali et al., 2022; J. D. Allen et al., 2022; K. Allen et al., 2022; Hnuploy et al., 2022; Holz et al., 2022; Kheil et al., 2022; Kitro et al., 2021; Liddell et al., 2021; Page et al., 2022; Reagu et al., 2023; Rego et al., 2022; Seo et al., 2022; Shaw et al., 2022; Talafha et al., 2022; West et al., 2021) |
| **COVID-19 Vaccine acceptance/intention proportion** | 32 (94%) | (Achangwa et al., 2021; Acharya et al., 2021; Aktürk et al., 2021; Al-Hatamleh et al., 2022; Alabdulla et al., 2021; Ali et al., 2022; J. D. Allen et al., 2022; K. Allen et al., 2022; Contreras-Pérez et al., 2022; Frisco et al., 2022; Führer et al., 2022; Hnuploy et al., 2022; Holz et al., 2022; Khaled et al., 2021; Kheil et al., 2022; Kitro et al., 2021; Liddell et al., 2021; Shen Lin, 2022; Martínez-Donate et al., 2022; Miner et al., 2023; Ogunbajo & Ojikutu, 2022; Reagu et al., 2023; Rego et al., 2022; Salibi et al., 2021; Seo et al., 2022; Shaw et al., 2022; Sudhinaraset et al., 2022; Talafha et al., 2022; Teng et al., 2023; Walker et al., 2021; West et al., 2021; Zhang et al., 2021) |
| **Knowledge/attitude/ beliefs towards Covid-19 vaccine** | 26 (76%) | (Achangwa et al., 2021; Acharya et al., 2021; Aktürk et al., 2021; Al-Hatamleh et al., 2022; Alabdulla et al., 2021; Ali et al., 2022; K. Allen et al., 2022; Contreras-Pérez et al., 2022; Frisco et al., 2022; Führer et al., 2022; Khaled et al., 2021; Kheil et al., 2022; Kitro et al., 2021; Lajunen & Wróbel, 2022; Liddell et al., 2021; Shen Lin, 2022; Martínez-Donate et al., 2022; Ogunbajo & Ojikutu, 2022; Rego et al., 2022; Salibi et al., 2021; Seo et al., 2022; Talafha et al., 2022; Teng et al., 2023; Walker et al., 2021; West et al., 2021; Zhang et al., 2021) |
| **Concern about COVID-19 vaccine safety/side effects** | 25 (74%) | (Achangwa et al., 2021; Acharya et al., 2021; Aktürk et al., 2021; Al-Hatamleh et al., 2022; Alabdulla et al., 2021; Ali et al., 2022; K. Allen et al., 2022; Contreras-Pérez et al., 2022; Frisco et al., 2022; Führer et al., 2022; Khaled et al., 2021; Kheil et al., 2022; Kitro et al., 2021; Lajunen & Wróbel, 2022; Liddell et al., 2021; Shen Lin, 2022; Martínez-Donate et al., 2022; Ogunbajo & Ojikutu, 2022; Salibi et al., 2021; Seo et al., 2022; Talafha et al., 2022; Teng et al., 2023; Walker et al., 2021; West et al., 2021; Zhang et al., 2021) |

| | | |
|---|---|---|
| **Concern about COVID-19 vaccine efficacy** | 17 (50%) | (Achangwa et al., 2021; Al-Hatamleh et al., 2022; Alabdulla et al., 2021; Ali et al., 2022; K. Allen et al., 2022; Contreras-Pérez et al., 2022; Führer et al., 2022; Kheil et al., 2022; Kitro et al., 2021; Lajunen & Wróbel, 2022; Liddell et al., 2021; Salibi et al., 2021; Seo et al., 2022; Talafha et al., 2022; Teng et al., 2023; Walker et al., 2021; Zhang et al., 2021) |
| **Concern about COVID-19 vaccine development** | 7 (21%) | (Aktürk et al., 2021; Alabdulla et al., 2021; Ali et al., 2022; Frisco et al., 2022; Ogunbajo & Ojikutu, 2022; Salibi et al., 2021; Talafha et al., 2022) |
| **Knowledge/attitude/ behavior regarding COVID-19 disease** | 20 (59%) | (Aktürk et al., 2021; Alabdulla et al., 2021; Ali et al., 2022; J. D. Allen et al., 2022; K. Allen et al., 2022; Contreras-Pérez et al., 2022; Frisco et al., 2022; Führer et al., 2022; Hnuploy et al., 2022; Holz et al., 2022; Khaled et al., 2021; Kitro et al., 2021; Liddell et al., 2021; Miner et al., 2023; Rego et al., 2022; Salibi et al., 2021; Talafha et al., 2022; Walker et al., 2021; West et al., 2021; Zhang et al., 2021) |
| **COVID-19 risk perception** | 15 (44%) | (Aktürk et al., 2021; Alabdulla et al., 2021; Ali et al., 2022; K. Allen et al., 2022; Contreras-Pérez et al., 2022; Frisco et al., 2022; Führer et al., 2022; Hnuploy et al., 2022; Holz et al., 2022; Khaled et al., 2021; Kitro et al., 2021; Liddell et al., 2021; Salibi et al., 2021; West et al., 2021; Zhang et al., 2021) |
| **History/ exposure to covid-19 in participants or their relations** | 16 (47%) | (Aktürk et al., 2021; Al-Hatamleh et al., 2022; Frisco et al., 2022; Hnuploy et al., 2022; Khaled et al., 2021; Kheil et al., 2022; Kitro et al., 2021; Shen Lin, 2022; Martínez-Donate et al., 2022; Ogunbajo & Ojikutu, 2022; Page et al., 2022; Rego et al., 2022; Talafha et al., 2022; Teng et al., 2023; West et al., 2021; Zhang et al., 2021) |
